# A machine learning approach to explore the spectra intensity pattern of peptides using tandem mass spectrometry data

**DOI:** 10.1186/1471-2105-9-325

**Published:** 2008-07-30

**Authors:** Cong Zhou, Lucas D Bowler, Jianfeng Feng

**Affiliations:** 1 Department of Informatics, University of Sussex, Brighton BN1 9QH, UK; 2 Sussex Proteomics Centre, TCMR, University of Sussex, Brighton BN1 9RY, UK; 3 Department of Computer Science and Mathematics, University of Warwick, Coventry CV4 7AL, UK; 4 Centre for Computational System Biology, Fudan University, Shanghai, PR China

## Abstract

**Background:**

A better understanding of the mechanisms involved in gas-phase fragmentation of peptides is essential for the development of more reliable algorithms for high-throughput protein identification using mass spectrometry (MS). Current methodologies depend predominantly on the use of derived m/z values of fragment ions, and, the knowledge provided by the intensity information present in MS/MS spectra has not been fully exploited. Indeed spectrum intensity information is very rarely utilized in the algorithms currently in use for high-throughput protein identification.

**Results:**

In this work, a Bayesian neural network approach is employed to analyze ion intensity information present in 13878 different MS/MS spectra. The influence of a library of 35 features on peptide fragmentation is examined under different proton mobility conditions. Useful rules involved in peptide fragmentation are found and subsets of features which have significant influence on fragmentation pathway of peptides are characterised. An intensity model is built based on the selected features and the model can make an accurate prediction of the intensity patterns for given MS/MS spectra. The predictions include not only the mean values of spectra intensity but also the variances that can be used to tolerate noises and system biases within experimental MS/MS spectra.

**Conclusion:**

The intensity patterns of fragmentation spectra are informative and can be used to analyze the influence of various characteristics of fragmented peptides on their fragmentation pathway. The features with significant influence can be used in turn to predict spectra intensities. Such information can help develop more reliable algorithms for peptide and protein identification.

## Background

Mass spectrometry (MS) has emerged in recent years as one of the most powerful tools for protein analysis available to proteomics research. MS-based protein identification strategies typically involve the digestion of protein samples prior to introduction into the mass spectrometer by a site-specific protease such as trypsin. The derived peptides are subsequently ionized at entry into the mass spectrometer and measured as intact fragment (parent) ions. Subsets of these ions can then be selected on the basis of their mass-to-charge ratio (m/z) and subject to further fragmentation, most commonly using collision induced dissociation (CID), in a process known as tandem mass spectrometry (MS/MS). Under the conditions utilized in CID, peptides fragment in predictable patterns resulting in a series of signature spectra. Identification of the protein components in an analyzed sample can then be achieved by correlating the observed signature spectra of individual peptides with the predicted MS/MS spectra of the amino acid sequences derived from protein databases such as Swiss-Prot and TrEMBL .

Over the past few years, computer-assisted database searching using mass spectrometry data has become the standard method for high-throughput protein identification. Unsurprisingly, the performance of computer search algorithms, for example Sequest , Mascot  and others, has a dramatic influence on the accuracy and reliability of the protein identification process [1]. In general terms, such algorithms use a built-in fragmentation model to construct theoretical fragmentation spectra for candidate peptides derived from databases, and then evaluate the match of these theoretical spectra with observed spectra from MS/MS experiments using defined scoring criteria. The candidate peptide whose predicted fragmentation spectra best matches the experimental MS/MS spectra is selected as representing the true identity of the analyzed peptide [2-5].

Unfortunately, the performance of computer algorithms currently available is still less than ideal. Generally, these algorithms tend to only utilize the positional information (mass-to-charge ratios; m/z) contained in MS/MS spectra, whereas fail to systematically incorporate the additional intensity information available in the same spectra. The intensity information is usually applied in an indirect way, for example in Mascot, peaks are selected based on intensity for peptide matching, and in Sequest peaks for y/b ions are supposed to be higher than peaks for other ions. Previous published work indicates that some efforts have been made to try to utilize spectrum intensity information more effectively [6-8], but they have predominantly focused on the design of better scoring methods. Furthermore, the application of these previous studies was limited by the oversimplification of the peptide fragmentation models which were applied to construct the theoretical spectra. This is probably due to our insufficient understanding of the complex mechanisms involved in peptide fragmentation during MS/MS analysis, which makes accurate prediction of spectra intensities in MS/MS very difficult.

Recently, a number of research groups have proposed novel fragmentation models in attempts to better understand the mechanisms involved in MS/MS. For example, Wysocki, *et al*. proposed the "Mobile Proton" hypothesis in which protons added to a peptide can transfer along its backbone from the initial site of protonation and subsequently induce fragmentation [9-11]. According to the hypothesis, peptides can be classified as "mobile" or "non-mobile" by the ratio of charge to Arginine number. They also statistically examined the effect of particular amino acid residues such as asparagine, proline and histidine on fragmentation patterns, with the aim of deducing rules for the influence of these residues on spectra intensities [12-14]. The "Mobile Proton" model was later expanded by Kapp, *et al*. into the "Relative Mobile Proton" (RMP) model [15], in which peptides are classified as "mobile", "non-mobile" and "partial mobile" based on their charge number and basic residue number. Schutz, *et al*. used a linear model based on RMP hypothesis to calculate the influence of sequence context effects on fragmentation [16]. A kinetic model was developed by Zhang [17,18] to simulate the fragmentation process of a peptide undergoing low-energy CID, and further used to predict the spectra intensity patterns of given peptides. Machine-learning techniques such as Bayesian decision trees have also been used to investigate peptide fragmentation behaviour [19] and from this work a group of features that may influence peptide fragmentation have been proposed. This was the first attempt to our knowledge to systematically utilize intensity information of peptide fragmentation. However, the machine learning approach used in [19] discovered only a limited number of features to have significant effect on peptide fragmentation and many of these features have already been revealed by other researchers, for example, the presence of basic residues in a peptide sequence, the charge state of the peptide and the presence of proline residue in peptide sequence, etc [10,11,20-22]. Whether many other putative determinants are of relevance, and the extent of their influence on fragmentation, is still in question.

In this work, we present a probabilistic machine-learning approach designed to analyze the intensity information contained in MS/MS data, with the aim of developing a better understanding of the rules involved in peptide fragmentation events. A library of peptide-relevant features as listed in Table 1 was examined and a score was assigned to each feature to represent the magnitude of its influence on peptide fragmentation. This information was then used to develop a more sophisticated model to predict the intensity patterns of spectra generated in MS/MS with the expectation that this will improve the reliability of peptide identification. Overall, we attempted to find answers for three basic questions: What factors influence peptide fragmentation during CID? What is the relationship between the features that influence peptide fragmentation and the resulting intensity pattern of fragmentation spectra? And finally, how can we accurately predict the spectrum intensity pattern of a given peptide and use this information to improve peptide identification?

**Table 1:** Features that potentially influence peptide fragmentation.

***ID***	***Features***	***Abbreviation***
1	Identity of residue C-terminal to fragmentation site	RB_C
2	Identity of residue N-terminal to fragmentation site	RB_N
3	Distance from fragmentation site to peptide N-terminus	DB_N
4	Distance from fragmentation site to peptide C-terminus	DB_C
5	Distance from fragmentation site to peptide center	DB_M
6	Whether fragmentation site is at either end of peptide	B_E
7	Basicity of residue N-terminal to fragmentation site	BaRB_N
8	Basicity of residue C-terminal to fragmentation site	BaRB_C
9	Average basicity of residues N/C terminal to fragmentation site	BaRB_A
10	Difference in basicity of residues N/C terminal to fragmentation site	BaRB_D
11	Basicity of fragmented y-ion	BaYI
12	Basicity of whole peptide	BaP
13	Helicity of residue N-terminal to fragmentation site	HeRB_N
14	Helicity of residue C-terminal to fragmentation site	HeRB_C
15	Average helicity of residues N/C terminal to fragmentation site	HeRB_A
16	Difference in helicity of residues N/C terminal to fragmentation site	HeRB_D
17	Hydrophobicity of residue N-terminal to fragmentation site	HyRB_N
18	Hydrophobicity of residue C-terminal to fragmentation site	HyRB_C
19	Average Hydrophobicity of residues N/C terminal to fragmentation site	HyRB_A
20	Difference in Hydrophobicity of residues N/C terminal to fragmentation site	HyRB_D
21	Hydrophobicity of fragmented y-ion	HyYI
22	Hydrophobicity of whole peptide	HyP
23	pI value of residue N-terminal to fragmentation site	PIRB_N
24	pI value of residue C-terminal to fragmentation site	PIRB_C
25	Average pI of residues N/C terminal to fragmentation site	PIRB_A
26	Difference in pI of residues N/C terminal to fragmentation site	PIRB_D
27	Length of whole peptide	LP
28	Length of fragmented y-ion	LYI
29	Ratio of length of fragmented y-ion and peptide	RLIP
30	Number of basic residues in whole peptide	NBaR_P
31	Number of basic residues in fragmented y-ion	NBaR_YI
32	Mass of whole peptide	MP
33	Mass of fragmented y-ion	MYI
34	Ratio of mass of fragmentated y-ion and peptide	RMIP
35	Distance from fragmentation site to basic residues	DBBa

All features listed above are supposed to exert influences on the gas-phase fragmentation of peptides. They are subject to further examination by the Bayesian neural network model.

## Results

The experimental design following development of our peptide fragmentation model comprised two phases: a feature selection stage for the determination of peptide characteristics that have significant influence on fragmentation, and a model development stage that trained a Bayesian neural network with features identified from the first stage. The performance of the model was then tested by using it to predict spectra intensity patterns for given peptides and subsequently compared with experimental data. Different data and data filtering algorithms were applied during the different phases.

### Experiment stage 1

**Figure 1:**
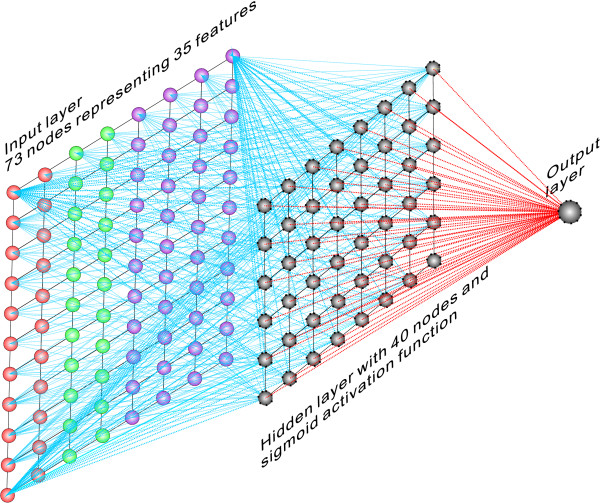
**Structure of the Bayesian neural network used to explore the mechanism of gas-phase fragmentation of peptides**. The network is fully connected and feed-forward with three layers including one hidden layer. 73 nodes are used in the input layer representing 35 features. 40 nodes in binary are used to represent the presence of 20 different residues at N and C terminus to the target peptide bond. Every node in the input layer has an independent coefficient to reveal its "relevance" to the network output. The hidden layer has 40 nodes and the activation function of the hidden layer is sigmoidal.

In this part of the study, MS/MS spectra data as described in [23] were acquired from Wysocki VH. The intensity information contained within the spectra was then used to verify a library of features that are supposed to influence peptide fragmentation (Table 1). The values of relevant amino acid properties that were used for calculating these features can be found in Table 2. This feature set is a modified version of what was used by Elias, *et al*. [19]. We aimed to determine a group of features that genuinely influence the intensity patterns of MS/MS spectra. For this purpose, a Bayesian neural network model was developed. The structure of the network model is illustrated in Figure 1 and more details can be found in the method section of the paper.

**Table 2:** Values of amino acid property used in the study.

**Residue**	**Mass**	**Hydrophobicity**	**Helicity**	**Basicity**	**PI value**
**A**	71.0788	0.16	1.24	206.4	6
**C**	103.1388	2.5	0.79	206.2	5.02
**D**	115.0886	-2.49	0.89	208.6	2.77
**E**	129.1155	-1.5	0.85	215.6	3.22
**F**	147.1766	5	1.26	212.1	5.48
**G**	57.0519	-3.31	1.15	202.7	5.97
**H**	137.1411	-4.63	0.97	223.7	7.47
**I**	113.1594	4.76	1.28	209.6	5.94
**K**	128.1741	-5	0.88	221.8	9.59
**L**	113.1594	4.76	1.28	209.6	5.98
**M**	131.1926	3.23	1.22	213.3	5.74
**N**	114.1038	-3.79	0.94	212.8	5.41
**P**	97.1167	-4.92	0.57	214.4	6.3
**Q**	128.1307	-2.76	0.96	214.2	5.65
**R**	156.1875	-2.77	0.95	237	11.15
**S**	87.0782	-2.85	1	207.6	5.68
**T**	101.1051	-1.08	1.09	211.7	5.64
**V**	99.1326	3.02	1.27	208.7	5.96
**W**	186.2132	4.88	1.07	216.1	5.89
**Y**	163.176	2	1.11	213.1	5.66

The values of different peptide property used in the study are listed in the table. Values for all the features listed in Table 1 are calculated with these property values during network training. The values for mass, hydrophobicity, helicity and basicity are cited from [19] and the values for PI are cited from

In brief the data comprised peptide MS/MS spectra from two micro organisms, *Shewanella oneidensis *and *Deinococcus radiodurans*. The datasets were derived using LC/MS analysis with ion trap instrumentation (further details can be found in the original paper [23]). Peptide sequences were assigned to these spectra using the Sequest algorithm with a minimum XCorr score of 1.5 for peptides with molecular weight < 1000, and 2.0 for all other peptides. Using the same chromatographic conditions, accurate masses of the precursor ions detected at the same retention time by FT-ICR were used to confirm the assigned sequences. Finally, a total of 28330 spectra of unique sequence and charge state (16008 from *Shewanella *and 12322 from *Deinococcus*) were acquired and subject to further analyses.

In our work, we wished to analyse only spectra with non-biased peptide intensities so that the genuine influences of all the features can be determined. For this purpose, the following filtering criteria were applied to the available 28330 spectra:

1. Only doubly charged peptide spectra were retained for the study.

2. For a given peptide, the intensities of detected b/y ions (plus ions resulting from degradation events) according to the assigned sequence, should be no less than 25% of the total intensities of all peaks within the particular spectrum. This criterion came from our belief that a correctly identified peptide should be able to explain all peaks in the corresponding spectra reasonably well. Accordingly all spectra with this correlation lower than an arbitrary threshold were considered to be either mismatches or biased spectra due to undetected degradation/modification events.

3. For a given peptide, the total intensities of the detected b/y ions should be no less than the intensity of the parent ion of the peptide. Application of this criterion was intended to ensure that all selected peptides are fully fragmented.

4. Finally, all candidate peptides were classified according to the "Relative Mobile Proton" (RMP) hypothesis [15]. Applying the RMP model as a classification criterion enables us to analyze peptides with different relative mobility separately, and also makes it easier for the machine learning algorithm to identify correct rules involved in peptide fragmentation.

As a result, a total number of 13878 spectra were analysed in this study, comprising 5768 mobile peptides, 7154 partially mobile peptides and 956 non-mobile peptides. The length of these peptides ranges from 5 to 40 with a mean value of 16. The data provided 208563 input patterns (peptide bonds) for the training of our network model.

**Figure 2:**
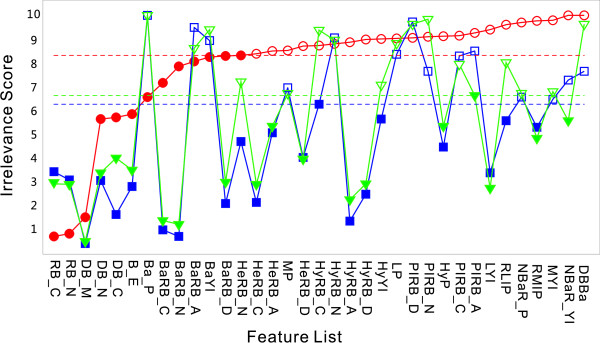
**Verification of the features that potentially influence peptide fragmentation**. The importance of the features listed in Table 1 is evaluated by the Bayesian neural network and the results are shown: Red circles: normalized irrelevance scores of the features under non-mobile status. Blue squares: normalized irrelevance scores of the features under partial-mobile status. Green triangles: normalized irrelevance scores of the features under mobile status. The higher an irrelevance score is, the less important the corresponding feature is. The threshold of each mobility status is shown in dashed line and the features proven to be influential on peptides' fragmentation (below threshold) are highlighted with filled circles/squares/triangles.

The first stage of our experiment, a feature selection stage as described above, began with training the Bayesian neural network 100 epochs using the features listed in Table 1. Details of network structure and training method can be found in the method section. The importance of each individual feature was evaluated by updating its "relevance coefficient" *α *as in Eq. 8. The results of coefficients were ranked and normalized, with their mean values defined as 'irrelevance' scores. The greater an irrelevance score is, the less significant the influence of the corresponding feature is. The irrelevance scores of each feature of different peptide mobility status are compared in Figure 2, and the values of the original scores can be found in Additional file 1.

As shown in Figure 2, the features that influence the fragmentation pathway of peptides vary considerably depending on peptide mobility status. Peptides of mobile or partial-mobile status generally share similar influential feature sets, but for peptides of non-mobile status, the features that influence fragmentation appear to be completely different. Such an observation implies that peptides of mobile- and partial-mobile status do not have fundamental differences in their fragmentation mechanism, whereas non-mobile peptides appear to possess their own unique method of fragmentation.

The results in Figure 2 indicate that the ion intensity pattern under non-mobile status depends highly on the sequence context of the fragmented peptide. It is well known that the identities of residues at the either side of a cleavage site play a very important role in determining whether cleavage can occur at this site, and the extent of this cleavage; but their influence are especially prominent for non-mobile peptides, who's spectra are often observed to be dominated by a limited number of ions. For mobile and partial-mobile peptides, however, fragmentation pathways appear to be determined by a mixture of factors including sequence context, position of cleavage site, mass and length of fragmented peptide, and many others.

The results show that cleavage is more likely to occur at the middle of a peptide rather than at the two ends, as mentioned before by Kapp, et al. [15,16]. We speculate that the specificity of tryptic digestion may contribute to this. It is also conceivable that the low mass cut-off inherent in ion trap mass spectrometers play a role in this position-selective phenomenon. It is observed that this phenomenon is less significant for non-mobile peptides, most likely because of the dominant residue-specific fragmentation pathway. Our analysis also reveals that the presence of basic residues can hinder fragmentation at peptide bonds close to them, as reported in other publications [14]. The influence of individual residue will be discussed in details in the next section.

It does not appear from our results that the basic nature of specific residues can influence the fragmentation pathway directly. Although the presence of basic residues within a peptide can result in marked changes in spectra intensity patterns, the basic nature of a particular residue (BaRB_N/C/A/D) appears to have little relevance to the fragmentation pattern (Figure 2). However, the basic characteristic of the whole peptide (BaP) does appear to play an important role in fragmentation irrespective of peptide mobility status, and the basic characteristics of fragmented y ions (BaYI) can influence peptides of mobile and partial-mobile status.

In general, the tendency of amino acid residues to contribute to the helicity nature of a peptide correlates with medium to high irrelevance scores, indicating that these characteristics do not have significant influence on peptide fragmentation, especially for non-mobile peptides (HeRB_N/C/A/D). Specific hydrophobicity-related features (HyRB_N, HyRB_C, HyYI), however, appear to be important in the fragmentation of both mobile and partial-mobile peptides, but they show little influence on peptides of non-mobile status. To the best of our knowledge there is no published theory suggesting a mechanism to explain how peptide hydrophobicity may influence fragmentation events in MS/MS, and, we are unsure whether our results stem from a causal relationship or simply a numerical correspondence. Indeed, this may be a topic worthy of future study. The PI values of residues show little direct influence on fragmentation of peptides (PIRB_N/C/A/D). The features "number of basic residues in the whole peptide" (NBaR_P) and "number of basic residues in the fragmentation ion" (NBaR_YI) were unsurprisingly ranked as having little influence on mobile peptides, because in the great majority of cases doubly charged mobile peptides contain only one single basic residue, which will be located at the C-terminus given the sequence specificity of trypsin. Accordingly these features are of little relevance for mobile peptides. In contrast, these two features do appear to be influential on peptides under other mobility status because variable numbers of basic residues are usually present in those cases. It is also apparent that the distance from the fragmented bond to basic residues has little influence on fragmentation pathways (DBBa). This feature indeed appears to influence mobile peptides, but such an effect is more likely to be a numerical correspondence only, because the sole basic residue in a doubly charged mobile peptide is located at its C-terminus, making this feature effectively synonymous with the feature "distance from fragmented bond to peptide C-terminus" (DB_C) that has been shown to influence peptide fragmentation. Finally, we find that the mass and length of fragmented ions/whole peptide can influence the overall fragmentation pattern (LP, LYI, MP, MYI). Comparatively, the ratio of mass/length are more influential (RLIP, RMIP) than absolute values of the two. This result agrees with findings reported elsewhere [24].

**Figure 3:**
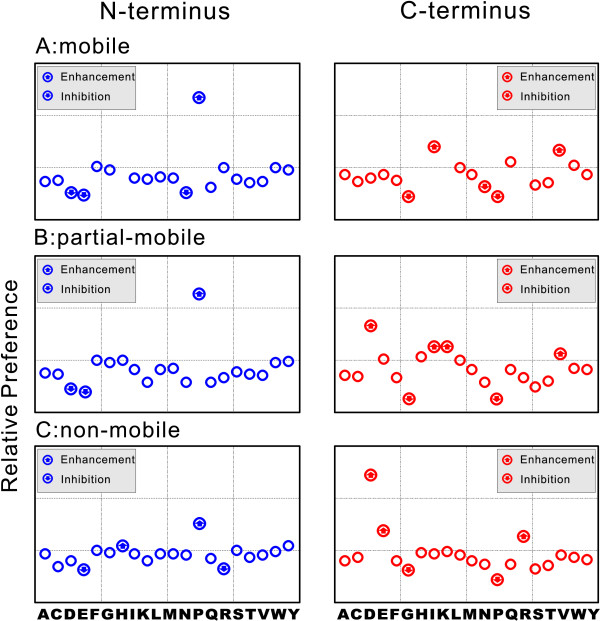
**Influence of each residue on fragmentation at its N/C terminal peptide bond**. The influence of each residue on cleavage at its N-terminus is illustrated in the left panel (blue dots), and the influence on cleavage at its C-terminus is illustrated in the right panel (red dots). The most influential residues are marked with arrows. Down arrows indicate inhibition whereas up arrows indicate enhancement. Figure 3-A: Mobile status. Figure 3-B: Partial-mobile status. Figure 3-C: Non-mobile status.

Many studies have been conducted to find out how the presence of a particular residue influences the subsequent fragmentation pathway of a whole peptide. A series of rules has been derived from both statistical analysis and manual interpretation of MS/MS spectra [9,12,14,16,17,19,23,24]. In our model, every residue has 2 separate nodes which represent its presence on the N- or C-terminus of a peptide bond. We are able to determine the influence of each residue by evaluating the weight values assigned to these nodes. The results are illustrated in Figure 3. As can be seen, many of the defined features appear to influence fragmentation, and most of them conform to the established rules. This correlation lends credence to the effectiveness of our approach, and supports the validity of the influence of the features as we suggest above.

When a free proton is available within a peptide (i.e. in a "mobile" peptide according to the RMP model), we unsurprisingly find that proline (P) has a significant influence on fragmentation. As has previously been extensively documented [9,14,16,17,19,23,24], proline markedly enhances cleavage at its N-terminal peptide bond while greatly inhibiting C-terminal cleavage. Conversely, aspartic acid (D) and glutamic acid (E) residues appear to inhibit cleavage at their N-termini, and similarly, asparagine (N) is found to have the same inhibitory effect on peptide fragmentation but to a less significant degree. Isoleucine (I) and valine (V) are found to promote C-terminal cleavage most, whereas glycine (G) and asparagine (N) residues have the greatest inhibitory effect (besides proline) on cleavage at the C-terminus.

However with non-mobile peptides, for example those containing multiple arginine (R) residues, protons are sequestered by the basic amino acids, and as a result the peptide fragments in a totally different manner (Figure 3-C). In this situation proline still has the greatest influence on cleavage on N- terminal cleavage, but in comparison to the situation in mobile peptides, this effect is much reduced. Aspartic acid is now the most influential residue in respect to enhanced C-terminal cleavage (as has been reported by many other researchers [12,16,17,23,24]), although its ability to inhibit cleavage at its N-terminal peptide bond is reduced. It is clear from the figure that the influence of aspartic acid is almost twice as much as that of proline, so even if they appear in the same peptide, the resulting spectra will be dominated by ions derived from aspartic acid-derived fragmentation. Glutamic acid (E) favours peptide cleavage at its C-terminus, a characteristic which probably results from the presence of a similar side chain to that of aspartic acid. Glycine-dependent inhibition of cleavage at its C-terminus is observed in all mobility status. Arginine (R) is observed to strongly promote cleavage at its C-terminus, and the other two basic residues Lysine (K) and Histidine (H) also present the same favour but in a less significant way. The rules defined above have also been reported previously by Wysocki group in work using a statistical method and the same MS/MS spectra dataset [23], and by Zhang, using his kinetic model [17,18].

We also observed a number of novel peptide sequence-context effects. Firstly arginine (R) residues show a markedly inhibited cleavage at their N-termini in non-mobile peptides. Secondly Histidine (H) appears to favour cleavage at its N-terminus, and such effect is observed in all mobility status. Besides these, previous studies have proposed that leucine (L) residues promote cleavage to their C-terminal peptide bonds, irrespective of the mobility status of the peptide [23]. This effect is not apparent from our study, with the presence of leucine only having a relatively minor effect (enhancement) on C-terminal cleavage (Figure 3.)

In the classical proton mobility theory peptides are classified into 2 distinct groups, designated as either mobile or non-mobile, according to the number of arginine residues present within the peptide. In addition, Kapp, *et al*. [15] have since proposed another class: an intermediate or 'partial' mobility state. We have also analyzed the fragmentation behaviour of peptides belonging to this third mobility class, and the results are indicated in Figure 3-B. We find that peptides falling into this notional group fragment according to a combination of rules predominant only to either mobile or non-mobile peptides. Effectively the mechanism of fragmentation in partially mobile peptides appears to obey a hybrid rule set. In this rule set, the influence of residues on fragmentation at their N-terminal peptide bonds is similar to that for peptides of mobile status, in which proline has a dominant enhancing effect, and aspartic acid and glutamic acid inhibit cleavage most. In marked contrast, the influence of amino acid residues on peptide bonds at their C-terminus more closely resembles that occurring in peptides of non-mobile status, where aspartic acid has the most profound effect. At the same time, isoleucine and valine enhance cleavage at their C-terminal peptide bonds in partial mobility peptides, as they do in mobile status peptides. Exceptionally, Lysine (K) is observed to enhance C-terminal cleavage in partially mobile peptides. Such an effect was not observed under any other mobility status. Glycine and proline have the most marked inhibitory effect on C-terminal cleavage as they do in both other peptide mobility groups.

It is worth noting that in the earlier work of Elias, *et al*. [19] using a Bayesian decision tree method, a similar feature set was examined to analyse the influence of each component on peptide fragmentation. In that study however, only the 'proline effect' was observed, and the influences of other residues were suggested to be insignificant. By revealing a considerably larger set of valid fragmentation rules using a similar feature set, it appears that our machine learning model has abetter learning capacity and is capable of identifying more subtle, yet nevertheless significant differences, in the contribution of different amino acid residues to peptide fragmentation during CID.

**Figure 4:**
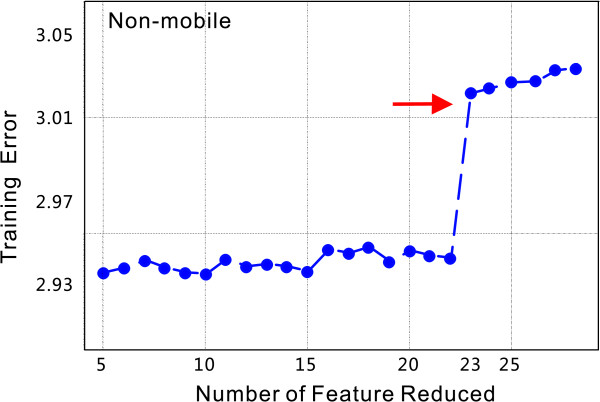
**Reduction of training errors in the feature selection phase**. Features are reduced according to their relevance to the fragmentation process (Figure 2). The X-axis represents the number of features being reduced and the Y-axis represents the average training error in percentage over 100 training times counted in percentage. The training error increases significantly when 23 less relevant features are removed, as indicated by the red arrow. It is then suggested that at most 22 features could be eliminated.

Having determined the irrelevance scores for all features examined, a new feature set can be defined containing only those found to markedly influence peptide fragmentation. To this end, we sequentially discarded the features with highest scores as listed in Figure 2, and then retrained the network with the reduced feature set. Comparison of the training results for all networks is illustrated in Figure 4. Taking non-mobile peptides as an example, the training error increases significantly when 23 less relevant features are removed, indicating that at most 22 features can be removed. The remaining features are indicated with filled circles in Figure 2.

### Experiment stage 2

With the reduced feature set derived in the first stage, a new network was trained to predict the intensity patterns of fragmentation spectra for given peptides. Benefiting from the application of the Bayesian theory, the network can not only predict the absolute values of spectra intensities, but also assign variances for the predictions. The obtained results are thus more robust against noise and system errors that unavoidably appear in the experimental MS/MS data. Details of the prediction method used can be found in the methods section.

A new MS/MS dataset was applied to evaluate the performance of the Bayesian intensity model. The dataset is a controlled dataset containing 18 different proteins as described in [25]. The details of how the dataset is generated can be found in the original paper. There are in total 1656 doubly charged spectra that have been verified to be correctly identified. Applying the same filtering method as described in experimental stage 1, we finally obtained 1607 doubly charged peptides for model testing. The theoretical spectra of these peptides were predicted by the Bayesian intensity model and then compared with the experimental counterparts to evaluate the accuracy of the model.

In order to compare an experimental spectrum with its predicted counterpart, a score capable of evaluating the similarity of two spectra has to be defined. As described in the method section, it is assumed that the log-transformed intensities of a given spectrum are Gaussian distributed with mean values and variances as predicted by our model. Accordingly, experimental spectra were normalized using the following method:

1. All peaks related to parent ions (for example 2+ parent ion and its degradations) are removed.

2. Divide each spectrum with its intensity of total ion current (TIC normalization) and then times 100.

3. Log-transformed and then normalized to [0, 1] scale.

It is then straightforward to design the following scoring system (Eq. 1) to measure the degree of similarity between the two spectra:

(1) $$Score=\sum_{k=1}^{n_{p}}\exp\left\{\frac{-{\left[I_{pk-predict}(w_{best})-I_{pk-real}\right]}^{2}}{2\sigma_{pk}^{2}}\right\}$$

where *n*_*p *_is the number of peptide bonds within peptide p, *I*_*pk*-*predict*_(*w*_*best*_) is the predicted mean intensity value of the peak at peptide bond *k*, *I*_*pk*-*real *_is the observed intensity of the peak at peptide bond *k *in the experimental spectrum, and *σ*_*pk *_is the standard deviation (SD) of the peak intensity at peptide bond *k *predicted by the intensity model. The more similar the two spectra, the higher the resulting score is.

**Figure 5:**
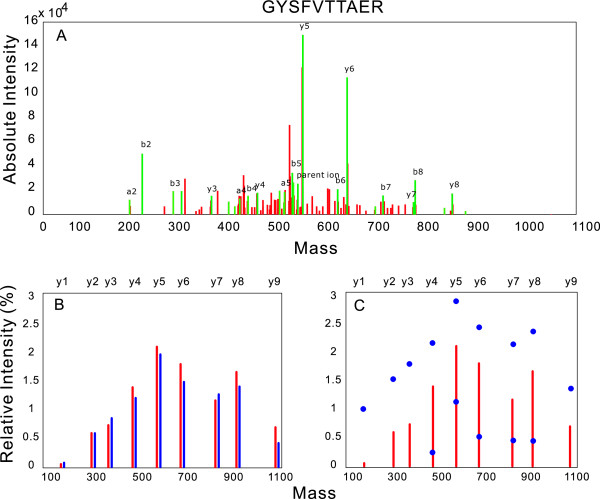
**Predicting spectra intensity pattern: on peptide GYSFVTTAER**. Figure 5-A: The raw MS/MS data of peptide GYSFVTTAER. Unlabeled green peaks are ions degraded from labeled b/y ions by losing H_2_O, NH_3_, etc. Figure 5-B: The comparison of the experimental spectrum (red) versus the spectrum predicted by the network model (blue). The experimental spectrum is the y-ions extracted from the raw data (Figure 5-A) with intensities log-transformed. Figure 5-C: The effect of using probability theory. Blue dots indicate the interval [mean intensity - SD, mean intensity + SD] within which intensities of the ions are supposed to lie.

An example of spectrum predicted by the Bayesian intensity model can be found in Figure 5 for the peptide GYSFVTTAER. The prediction for this peptide achieves one of the highest scores (best fit). It can be seen that the predicted spectrum matches its experimental counterpart very well, and the small differences between the two spectra are well within the variance. Further examples using peptides of different mobility status can be found in Additional file 2.

**Figure 6:**
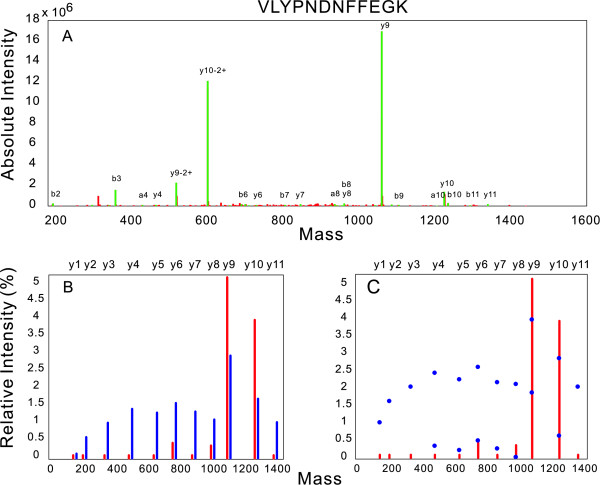
**Predicting spectra intensity pattern: on peptide VLYPNDNFFEGK**. Figure 6-A: The raw MS/MS data of peptide VLYPNDNFFEGK. Unlabeled green peaks are ions degraded from labeled b/y ions by losing H_2_O, NH_3_, etc. Figure 6-B: The comparison of the experimental spectrum (red) versus the spectrum predicted by the network model (blue). The experimental spectrum is the y-ions extracted from the raw data (Figure 6-A) with intensities log-transformed. Figure 6-C: The effect of using probability theory. Blue dots indicate the interval [mean intensity - SD, mean intensity + SD] within which intensities of the ions are supposed to lie.

Similarly, the prediction for peptide VLYPNDNFFEGK is illustrated in Figure 6. This peptide attained one of the lowest scores (worst fit), indicating a probable failure of spectrum prediction. Indeed, as can be seen in Figure 6-C, none of the peaks lie within the expected ranges. It is apparent from Figure 6-A that the experimental spectrum of the peptide is dominated by the y9 ion resulting from cleavage at the Y-P bond of the peptide, the other ions of the expected y-ion series are either very low or even below the level of detection. This pattern is characteristic of the type of spectrum that often leads to random (false) matches in database searching using current m/z based peptide identification algorithms. However, as shown in Figure 6-B, our model did correctly predict the general pattern of the spectrum, i.e. that y9 and y10 are the highest two peaks and the others peaks are lower and of relatively equal height. The experimental spectrum therefore represents a greatly exaggerated version of the predicted pattern.

**Figure 7:**
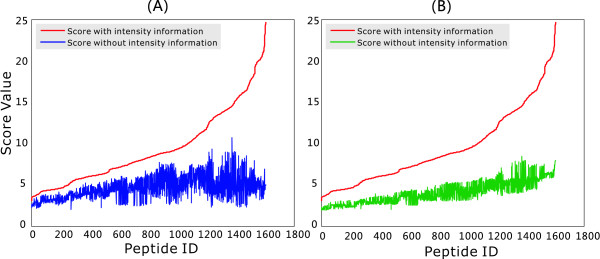
**Comparison of scores with/without intensity information on all test peptides**. Similarity scores are computed using Eq. 1. The higher a score, the more similar the predicted spectrum is to the experimental counterpart. Figure 7-A: The red line represents the sorted scores calculated with the predicted intensity information. The blue line represents the corresponding scores calculated without intensity information. The two score use the same variances predicted by the Bayesian neural network model. Figure 7-B: The red line represents the sorted scores calculated with the predicted intensity information. The blue line represents the corresponding scores calculated without intensity information. Variances for intensity-free scores are set to 1.

The similarity scores were firstly calculated for spectra predicted by the Bayesian intensity model as illustrated in Figure 7. The same scores were subsequently recalculated with intensity information excluded, i.e. after assigning the same intensity value to each peak within a spectrum. Such an approach, using intensity-free spectra, is typical of most peptide identification algorithms in current use, e.g. Sequest. In order to compare spectra with/without intensity information on an impartial basis, the similarity scores for intensity-free spectra were firstly calculated using the same variance values as used for scores with intensity information, and then recalculated with the influence of variance eliminated (set *σ*_pk _= 1). The former case was illustrated in Figure 7-A and the latter one in Figure 7-B. It can be clearly seen that the scores derived using intensity information are consistently higher than those derived without. This result indicates that our network model can accurately predict fragmentation spectra for given peptides, and the predicted spectra fit experimental spectra much better than those generated using intensity-free information.

In order to further validate our Bayesian intensity model, programs for kinetic intensity model published by Zhang [17] were used to predict the intensity patterns of the same test dataset as mentioned above. The differences between experimental spectra and predicted counterparts are calculated and compared with those from our Bayesian intensity model. It should be noted that Zhang's kinetic model is able to predict intensity of b, y ions and their degradations. So only y related ions were pick out for comparison. At the same time, the variance information predicted by the Bayesian model was also ignored. As listed in Table 3, the Bayesian model has a smaller prediction error in 897 spectra out of the total 1607, showing a slightly higher accuracy than the kinetic model. However, the mean values and SD values of prediction errors from the two approaches are rather close. It is reasonable to conclude that the two approaches have similar performance in predicting spectra intensity values for given peptides, and our Bayesian intensity model can potentially be more informative because extra variances can be assigned to the predictions to tolerate the prediction error.

**Table 3:** Prediction error of the kinetic model and the Bayesian intensity model.

	**Number of spectra with higher accuracy**	**Mean error**	**SD error**
**Kinetic model**	710	0.4294	0.1769
**Bayesian intensity model**	897	0.4094	0.1650

The intensity patterns of 1607 test data are predicted with Zhang's kinetic model and our Bayesian intensity model. The accuracy of predictions and mean/SD of prediction errors are listed in the table.

## Discussion

In this work, a novel Bayesian neural network approach was applied to examine features that were thought to influence peptide fragmentation. The benefit of this approach includes making the features numerically analysable so that large number of features regarding various characteristics of fragmented peptides can be compared directly at one time. In the experiment stage 2, a new network was trained to predict intensity patterns of fragmentation spectra for given peptides. Only a limited number of features with significant influence were applied in this stage, and the others were discarded. It is worth noting that the discarded features are not necessarily irrelevant to peptide fragmentation. Indeed, they may still influence fragmentation pathways but in a less significant or indirect way. However they were discarded given that results indicated that the accuracy of spectra intensity predictions was not significantly affected by the elimination of these features.

The MS/MS data used in this work is dominated by spectra from mobile and partial-mobile peptides, whereas the number of non-mobile data is relatively small. This is mainly because these peptides were identified by Sequest, who rely heavily on m/z information to make identification. Non-mobile peptides, unfortunately, usually fail to present enough amounts of ions in the spectra, and therefore fail to be identified. It is worth noting that although many reasonable rules in fragmentation have been derived for non-mobile peptides, it is very likely that some important fragmentation rules are missing.

To the best of our knowledge, most algorithms available for peptide identification to date make their identification based on spectra m/z information only. The importance of spectra intensity information on peptide identification have been realised by many researchers, but successful applications in published literature are still rare: [26] is the only one to the best of our knowledge, and it is simply an application of Zhang's kinetic model. This phenomenon is partially because it is difficult to predict intensity patterns of fragmentation spectra accurately, and even if intensity patterns are successfully predicted, how to compare the predicted spectra with experimental ones still remains a problem. The large volumes of noise and system errors inevitably contained in experimental spectra make it difficult to apply conventional comparison methods to evaluate the true degree of similarity between spectra. Our network model provided a good way to solve the problem by assigning variances to the predictions to obtain certain degree of tolerance to the fluctuation of spectra intensities. In this study, we proposed a scoring method (Eq. 1) that combined the predicted variance information to compare two spectra under total ion current normalization. This method worked well in validating the intensity model and could be used for peptide identification. However, there were still cases in which the predicted spectra failed to match the experimental counterpart even if the general pattern of spectra intensity was predicted correctly (Figure 6). Future work will involve development of more robust scoring methods to further improve the performance of our intensity model in peptide identification, and allow for the effect of post-translational modifications (PTMs) on peptide fragmentation pathways. The influence of PTMs is unpredictable at present, as modified peptides may fragment in a different manner to unmodified ones, thereby making predictions using current fragmentation models less reliable.

It is important to note that the intensity pattern of MS/MS spectra for the same peptide can differ depending on the nature of the mass spectrometer platform used for analysis, e.g. whether it is an ion trap or a Q-ToF system etc., and the method of ionisation employed (e.g. electrospray or MALDI etc.). In this situation, the intensity model needs to be retrained each time to adapt to the different machine types and peptide dissociation methods (e.g. CID, electron capture dissociation or electron transfer dissociation)

## Conclusion

In this work, we have shown that the intensity patterns of fragmentation spectra are informative and can be used to analyze the influence of various characteristics of fragmented peptides on their fragmentation pathway. The features with significant influence can be used in turn to predict spectra intensities given the sequences of peptides. It has been demonstrated that the intensity pattern of fragmentation spectra predicted by our model fits experimental data reasonably well. It is suggested that such intensity predictions can be used with current peptide and protein identification algorithms to make them more reliable in high-throughput proteomics experiments.

## Methods

We proposed that ion intensities resulting from peptide fragmentation in MS/MS can be expressed as a complicated mathematical function of various features that reflect the physical, chemical and other characteristics of fragmented peptides. Accordingly, a Bayesian neural network was constructed in an attempt to link the spectrum intensity of a peptide and these features together. The Automatic Relevance Determination (ARD) technique [27,28] was applied to this network to distinguish features (input) that significantly influence intensity patterns of fragmentation spectra (output) from those that do not. The structure of the Bayesian neural network used in the study is shown in Figure 1. The network is a fully connected feed-forward neural network comprising three layers: an input layer, a hidden layer and an output layer. In our study, we used this network to analyse a library of features that were supposed to influence peptide fragmentation, distinguishing those with significant influence from non-influential or less-influential ones. The feature set used in our study is a modified version of what was used by Elias, *et al*. [19]. The original feature set was reduced by eliminating features that unfit for our study, such as b/y ion type and identity of residues at N/C terminus of peptides. The descriptions and abbreviations used for the features applied in our study are listed in Table 1, and the values of relevant amino acid properties that were used for calculating these features can be found in Table 2.

In this study, we considered all bonds within a given peptide to represent potential sites for fragmentation. As a consequence, the failure to observe peaks resulting from cleavage at these sites were not considered to denote that cleavage had not occurred at these peptide bonds, but was instead taken to indicate that cleavage at the sites concerned was poor, i.e. the relevant peaks were too low in intensity to be separated from the background noise. Accordingly, all bonds within a given peptide were coded separately into the network. One set of input corresponded to features derived from only one target peptide bond. We used 73 nodes in the input layer of the Bayesian neural network to represent the 35 features of target peptide/peptide bond as listed in Table 1. In order to represent the identification of the residue at N- or C- terminus of the target peptide bond, we used 20 nodes to cover all the 20 alternative amino acid identifications of one residue. Their values are binary such that only one of the 20 nodes that correspond to the identification of the target residue was set to 1 during training and all others were set to zero. Every node in the input layer has been given an independent coefficient to reveal its "relevance" to the network output. The hidden layer comprises 40 nodes, making the network less complicated while simultaneously maintaining enough computational power. The activation function of the hidden layer is sigmoidal: *f *(*x*) = 1+e^-*ωx*^, where *ω *is the parameter used to control saturation. Finally, the neural network has only one output, defined to represent the quantity of ions generated from fragmentation at the specific peptide bond, i.e. it represents the unnormalized spectrum intensity of the particular target peptide bond. The network and all the other relevant programs were implemented using Matlab.

Theoretically, one peptide can fragment into a complete series of ions of smaller mass, comprising those designated as x, y, z and a, b, c ions, among which b and y ions are usually the most prominent [20,24]. Typically, classical protein identification algorithms rank peptide candidates according to the number of matches for both the b and y ion series. However, it has been observed that b ions are more likely to degrade into a variety of ions with lower mass by losing CO, NH_3 _or other neutral components [24], thus introducing difficulties in accurate evaluation of their original intensities. To allow us to take advantage of the intensity information of spectra, we made the assumption that a doubly charged peptide can only fragment into two singly charged ions rather than (albeit relatively rarely), a doubly charged ion and a neutral counterpart. Under this assumption, y/b ions would be generated at the same rate during fragmentation, and thus manifest the same ion intensities (before degradation) on MS/MS maps, enabling us to focus only on intensities of the y ions. In practice, however, doubly charged y ions were still taken into account. We recorded the ion intensity of a certain fragmentation site by summing up the intensities of all y-related ions, including singly and doubly charged y-ions and intensities of their degraded ions from losses of H_2_O and NH_3_, while disregarding the intensities of the complementary b-ion series. Recorded intensity values of a peptide were firstly normalized by dividing its intensity of total ion current and then times 100 to unify different intensity magnitude among peptides. They are subsequently subject to log transformation to reduce intensity-dependent variances [29] and finally normalized to [0, 1] scale.

For the *p*^*th *^peptide with *n*_*p *_peptide bonds, we have an input set {*B*_*p*1_, *B*_*p*2_,..., $B_{pn_{p}}$} representing all bonds within the peptide. We obtain a set of output {*O*_*p*1_,*O*_*p*2_,..., $O_{pn_{p}}$} from the neural network that can be normalized to a relative scale, as shown in Eq. 2:

(2) *I*_*pk*-*predict *_= 100·*O*_*pk*_/O_*pmax*_   (k = 1,⋯, n_p_)

where *O*_*pmax *_= *max*{*O*_*p*1_, *O*_*p*2_,..., $O_{pn_{p}}$}. The normalized output set *I*_*pk*-*predict *_(*k *= 1,..., *n*_*p*_) can hence be viewed as normalized spectra intensities to approximate the real spectra intensities *I*_*pk*-*real *_(*k *= 1,..., *n*_*p*_) observed from experimental MS/MS data.

In contrast to the classical back-propagation algorithm [27], the normalization process used in the training of the neural network, leads to a unique way of updating network weights. Let *E*_*pk *_be the error calculated at the *k*^*th *^peptide bond of the *p*^*th *^peptide,

*E*_*pk *_= (*I*_*pk*-*predict *_- *I*_*pk*-*real*_)^2^/2

the derivatives of *E*_*pk *_can be evaluated using

(3) $$\frac{dE_{pk}}{dW_{ij}}=\left(I_{pk-predict}-I_{pk-real}\right)\cdot \frac{100}{O_{pmax}}\cdot \left(\frac{dO_{pk}}{dW_{ij}}-\frac{O_{pk}}{O_{\max}}\cdot \frac{dO_{pmax}}{dW_{ij}}\right)$$

where *W*_*ij *_is the weight connecting node *i *and *j*.

A Bayesian inference is applied with the neural network by assuming that weights of the neural network *W *= {*W*_*ij*_} have a Gaussian prior distribution [26,27]:

(4) $$P(W)=\frac{1}{Z_{W}(\alpha )}\cdot \exp\left(-\alpha \cdot E_{W}\right)$$

where *α *is the parameter controlling the distribution of weights, *Z*_*W*_(*α*) is a normalization constant, and E_W _is the error function for the weights defined as: *E*_*W *_= ||*W*||^2^/2. We have also assumed that the noise present in experimental MS/MS data also has a Gaussian prior distribution,

(5) $$P(D|W)=\frac{1}{Z_{D}(\beta )}\cdot \exp\left(-\beta \cdot E_{D}\right)$$

where *D *is the experimental data, *β *is the parameter controlling the distribution of noise, *Z*_*D*_(*β*) is a normalization constant, *E*_*D *_is the summation of error for all training peptide bonds:

$$E_{D}=\sum_{p=1}^{m}\sum_{k=1}^{n_{p}}E_{pk}$$

where *m *is the total number of peptides used in network training and *n*_*p *_is the number of bonds in the *p*^*th *^peptide. By applying Bayesian theory on Eq. 4, Eq. 5, we have:

(6) $$P(W|D)=\frac{P(D|W)\cdot P(W)}{P(D)}\propto \exp\left(-\beta E_{D}\cdot \alpha E_{W}\right)$$

We need to maximize *P(W*|*D) *to train the neural network, which is equal to minimize

(7) *E *= *β*·*E*_*D *_+ *α*·*E*_*W *_

where *E*_*D*_, *E*_*W *_are error evaluated for network output and weights as defined before, parameters *α*, *β *are pre-set values donating our guess on distribution of weights and noise. The value of *α *and *β *are periodically re-evaluated during training by the equation:

(8) $$\begin{array}{cc} \alpha =\frac{Y}{2\cdot E_{W}}, & \beta =\frac{\sum_{p=1}^{m}n_{p}}{2\cdot E_{D}} \end{array}$$

thereby updating our initial estimates regarding the distribution of weights and noise, where *Y *is the dimension of weights in the network.

In our network, every input node has a separate parameter a to control distribution of weights connecting to it. According to the ARD theory [27,28], *α *can also be used to evaluate the relevance of each input to output. In practice, we trained the neural network 100 times with random initial values of weights, and then evaluated *α *for each input feature by taking the normalized average over all training loops. The processed values of *α*, denoted as irrelevance scores, are illustrated in Figure 2 and are discussed in the experimental section. A set of features that are most relevant to peptide fragmentation can be acquired by gradually reducing less important features in the input (Figure 4).

Using selected features that have been proven to have significant influence on peptide fragmentation, another Bayesian neural network was constructed to predict the intensity of fragmentation spectra given the sequence of a peptide. Such prediction has long been recognized as a difficulty because MS/MS data typically contains a large volume of noise. This noise results from a variety of factors, most of which are not relevant to the fragmentation pathway followed by the peptide itself, including differences in sample preparation methodologies, the ionization method used, the type of mass spectrometer, etc. Even under identical experimental conditions, the ion intensities resulting from fragmentation of a particular peptide bond can vary considerably from experiment to experiment, making accurate prediction impossible. Accordingly, it is reasonable to take the fragmentation process as a random event, i.e. although the fragmentation pathway of a given peptide is invariable, the relative ion intensities generated along the pathway are not fixed. For each potential ion-type, the degree of fragmentation can fluctuate by a limited but nevertheless significant amount, whereas for its MS/MS map, the relative ion intensities values may vary considerably because of normalization. In this work, we assumed that the quantity of each ion species is Gaussian distributed. This assumption was applied by assigning variances to outputs of the new network with the reduced input feature set. For the *k*^*th *^bond within the *p*^*th *^unknown peptide, normalization process (Eq. 2) becomes a linear transformation. We have:

(9) $$\begin{array}{c} P\left(O_{pk}|B_{pk},D\right)=\int P\left(O_{pk}|B_{pk},W\right)\cdot P\left(W|D\right)dW\\ \propto \int P\left(D|W\right)\cdot P\left(W|D\right)dW \end{array}$$

where *P*(*W*|*D*) is the posterior distribution of network weights defined by Eq. 6. By applying the Taylor expansion on training error (Eq. 7) around the weights whose values maximize (locally) *P*(*W*|*D*) and retain terms up to second order, we have

(10) $$E=E\left(W_{best}\right)+\frac{1}{2}\cdot {\left(W-W_{best}\right)}^{T}\cdot H\cdot \left(W-W_{best}\right)$$

where *W*_*best *_is the weights that maximize (locally) *P*(*W*|*D*) and *H *is the Hessian matrix of error function. By applying Eq. 5 and Eq. 10 on Eq. 9, we have

(11) $$\begin{array}{l} P\left(O_{pk}|B_{pk},D\right)\propto \int P\left(D|W\right)\cdot P\left(W|D\right)\;dw\\ \propto \int \exp\left(-\beta \cdot E_{pk}-\frac{1}{2}\cdot {\left(W-W_{best}\right)}^{T}\cdot H\cdot \left(W-W_{best}\right)\right)dw \end{array}$$

We then approximate *I*_*pk*-*predict *_by its linear expansion around *W*_*best *_(as defined before),

(12) *I*_*pk*-*predict *_= *I*_*pk*-*predict *_(*W*_*best*_) + *g*^*T*^(*W *- *W*_*best*_)

where *g *is the first derivative of *I*_*pk*-*predict*_. By applying Eq. 12 on Eq. 11,

(13) $$\begin{array}{l} P\left(O_{pk}|B_{pk},D\right)=\frac{1}{{\left(2\pi \sigma_{pk}^{2}\right)}^{1/2}}\cdot \exp\left\{-\frac{{\left[I_{pk-predict}(W_{best})-I_{pk-real}\right]}^{2}}{2\sigma_{pk}^{2}}\right\}\\ \sigma_{pk}^{2}=\frac{1}{\beta }+g^{T}\cdot H^{-1}\cdot g \end{array}$$

where *β *is the parameter defined in Eq. 5, Eq. 8. Eq. 13 informs us that the unnormalized spectra intensities predicted by our neural network are actually Gaussian distributed with mean values directly given by the output and variances given by Eq. 13 revealing that variances come from two factors: the average noise level contained in the MS/MS training data and the characteristics of the particular cleavage peptide bond.

## Authors' contributions

CZ: Carried out the main data analysis work and the writing of the manuscript. LDB: Mass spectrometry: Generation of MS/MS data and participation in writing the manuscript. JF: Study design and overall supervision of the project.

## Competing interests

The authors declare that they have no competing interests.

## Supplementary Material

Additional file 1 Original Values of irrelevance score for all features. The original values of irrelevance scores for all features are listed in the file. Click here for file

Additional file 2 Examples of spectra predicted by the Bayesian neural network model. Spectra with different peptide mobilities are predicted by the Bayesian neural network model and illustrated in the file. Click here for file

## References

[B1] Chamrad DC, Korting G, Stuhler K, Meyer HE, Klose J, Bluggel M (2004). Evaluation of algorithms for protein identification from sequence databases using mass spectrometry data. Proteomics.

[B2] Eng JK, McCormack AL, Yates JR (1994). An Approach to Correlate Tandem Mass Spectra Data of Peptides with Amino Acid Sequences in a Protein Database. J Am Soc Mass Spectrom.

[B3] Perkins DN, Pappin DJ, Creasy DM, Cottrell JS (1999). Probability-based protein identification by searching sequence databases using mass spectrometry data. Electrophoresis.

[B4] Sadygov RG, Yates JR (2003). A hypergeometric probability model for protein identification and validation using tandem mass spectral data and protein sequence databases. Anal Chem.

[B5] Fenyo D, Beavis RC (2003). A method for assessing the statistical significance of mass spectrometry-based protein identifications using general scoring schemes. Anal Chem.

[B6] Havilio M, Haddad Y, Smilansky Z (2003). Intensity-based statistical scorer for tandem mass spectrometry. Anal Chem.

[B7] Bern M, Goldberg D, McDonald WH, Yates JR (2004). Automatic quality assessment of peptide tandem mass spectra. Bioinformatics.

[B8] Narasimhan C, Tabb DL, Verberkmoes NC, Thompson MR, Hettich RL, Uberbacher EC (2005). MASPIC: intensity-based tandem mass spectrometry scoring scheme that improves peptide identification at high confidence. Anal Chem.

[B9] Wysocki VH, Tsaprailis G, Smith LL, Breci LA (2000). Mobile and localized protons: a framework for understanding peptide dissociation. J Mass Spectrom.

[B10] Dongré AR, Jones JL, Somogyi Á, Wysocki VH (1996). Influence of peptide composition, gas-phase basicity, and chemical modification on fragmentation efficiency: evidence for the mobile proton model. J Am Soc Mass Spectrom.

[B11] Tsaprailis G, Nair H, Somogyi Á, Wysocki VH, Zhong W, Futrell JH, Summerfield SG, Gaskell SJ (1999). Influence of secondary structure on the fragmentation of protonated peptides. J Am Chem Soc.

[B12] Gu C, Tsaprailis G, Breci L, Wysocki VH (2000). Selective gas-phase cleavage at the peptide bond terminal to aspartic acid in fixed-charge derivatives of asp-containing peptides. Anal Chem.

[B13] Tsaprailis G, Nair H, Zhong W, Kuppannan K, Futrell JH, Wysocki VH (2004). A mechanistic investigation of the enhanced cleavage at histidine in the gas-phase dissociation of protonated peptides. Anal Chem.

[B14] Breci LA, Tabb DL, Yates JR, Wysocki VH (2003). Cleavage N-terminal to proline: analysis of a database of peptide tandem mass spectra. Anal Chem.

[B15] Kapp EA, Schutz F, Reid GE, Eddes JS, Moritz RL, O'Hair RA, Speed TP, Simpson RJ (2003). Mining a tandem mass spectrometry database to determine the trends and global factors influencing peptides' fragmentation. Anal Chem.

[B16] Schutz F, Kapp EA, Simpson RJ, Speed TP (2003). Deriving statistical models for predicting peptide tandem MS product ion intensities. Biochem Soc Trans.

[B17] Zhang Z (2004). Prediction of low-energy collision-induced dissociation spectra of peptides. Anal Chem.

[B18] Zhang Z (2005). Prediction of low-energy collision-induced dissociation spectra of peptides with three or more charges. Anal Chem.

[B19] Elias JE, Gibbons FD, King OD, Roth FP, Gygi SP (2004). Intensity-based protein identification by machine learning from a library of tandem mass spectra. Nature Biotechnology.

[B20] Graves PR, Haystead TAJ (2002). Molecular Biologist's Guide to Proteomics. Microbiology and Molecular Biology Reviews.

[B21] Tabb DL, Huang Y, Wysocki VH, Yates JR (2004). Influence of basic residue content on fragment ion peak intensities in low-energy collision-induced dissociation spectra of peptides. Anal Chem.

[B22] Savitski MM, Kjeldsen F, Nielsen ML, Garbuzynskiy SO, Galzitskaya OV, Surin AK, Zubarev RA (2007). Backbone Carbonyl Group Basicities Are Related to Gas-Phase Fragmentation of Peptides and Protein Folding. Angew Chem Int Ed Engl.

[B23] Huang Y, Triscari JM, Tseng GC, Pasa-Tolic L, Lipton MS, Smith RD, Wysocki VH (2005). Statistical Characterization of the Charge State and Residue Dependence of Low-Energy CID Peptide Dissociation Patterns. Anal Chem.

[B24] Tabb DL (2003). Statistical characterization of ion trap tandem mass spectra from doubly charged trypic peptides. Anal Chem.

[B25] Keller A, Purvine S, Nesvizhskii A, Stolyar S, Goodlett DR, Kolker E (2002). Experimental Protein Mixture for Validating Tandem Mass Spectral Analysis. OMICS.

[B26] Sun S, Meyer-Arendt K, Eichelberger B, Brown R, Yen C-Y, Old WM, Pierce K, Cios KJ, Ahn NG, Resing KA (2007). Improved validation of peptide MS/MS assignments using spectral intensity prediction. Mol Cell Proteomics.

[B27] Bishop CM (1995). Neural networks for pattern recognition. Clarendon Press/OUP.

[B28] MacKay D (1995). Bayesian methods for neural networks: theory and applications. Course notes for Neural Networks Summer School.

[B29] Sauve AC, Speed TP (2004). Normalization, Baseline Correction and Alignment of High-throughput Mass Spectrometry Data. Data proceedings Gensips.

