# Supplementary material for: A machine learning approach to explore the spectra intensity pattern of peptides using tandem mass spectrometry data
Source: BMC Bioinformatics. 2008 Jul 30;9:325. doi: 10.1186/1471-2105-9-325 (PMC2529326; doi:10.1186/1471-2105-9-325)
Supplement: Additional file 1 — Original Values of irrelevance score for all features. The original values of irrelevance scores for all features are listed in the file. [file 1471-2105-9-325-S1.pdf]

Table 1 – Original Values of irrelevance score for all features:

Abbreviation	*Mobile*	*Partial*	*Nonmobile*	*Abbreviation*	*Mobile*	*Partial*	*Nonmobile*
RB_C	0.44	10.21	1.65	HyRB_A	4.51	28.44	20.64
RB_N	0.53	11.40	1.35	HyRB_D	3.46	21.95	22.38
DB_N	4.81	30.44	18.81	HyYI	1.35	11.23	21.51
DB_C	1.49	6.81	18.94	HyP	3.37	28.93	21.17
DB_M	0.19	0.98	3.24	PIRB_N	4.79	21.19	19.96
B_E	2.44	17.89	22.35	PIRB_C	4.05	28.21	20.92
BaRB_N	5.04	26.00	20.80	PIRB_A	4.58	30.81	20.08
BaRB_C	4.40	32.35	18.37	PIRB_D	5.12	34.14	14.91
BaRB_A	4.93	33.14	20.69	LP	1.68	10.16	12.81
BaRB_D	4.93	26.02	22.90	LYI	1.44	6.93	19.15
BaYI	1.46	8.14	20.52	RLIP	0.56	2.04	17.90
BaP	1.75	9.26	13.25	NBaR_P	3.67	15.79	18.97
HeRB_N	3.40	23.61	19.47	NBaR_YI	4.09	18.79	21.96
HeRB_C	1.98	13.47	19.86	MP	2.02	5.25	12.93
HeRB_A	3.60	19.05	20.60	MYI	1.11	4.26	20.25
HeRB_D	3.42	22.25	22.17	RMIP	0.65	3.02	16.32
HyRB_N	2.70	15.04	20.85	DBBa	2.82	24.68	22.87
HyRB_C	2.70	17.07	19.43				

The original values of irrelevance scores for all features are listed above. The higher a score, the less important the corresponding feature is. Features of no apparent significance to peptide fragmentation are indicated in green and are eliminated in experiment stage 2.
