# Supplementary material for: A machine learning approach to explore the spectra intensity pattern of peptides using tandem mass spectrometry data
Source: BMC Bioinformatics. 2008 Jul 30;9:325. doi: 10.1186/1471-2105-9-325 (PMC2529326; doi:10.1186/1471-2105-9-325)
Supplement: Additional file 2 — Examples of spectra predicted by the Bayesian neural network model. Spectra with different peptide mobilities are predicted by the Bayesian neural network model and illustrated in the file. [file 1471-2105-9-325-S2.pdf]

Figure 1 – Examples of spectra predicted by the Bayesian neural network model:

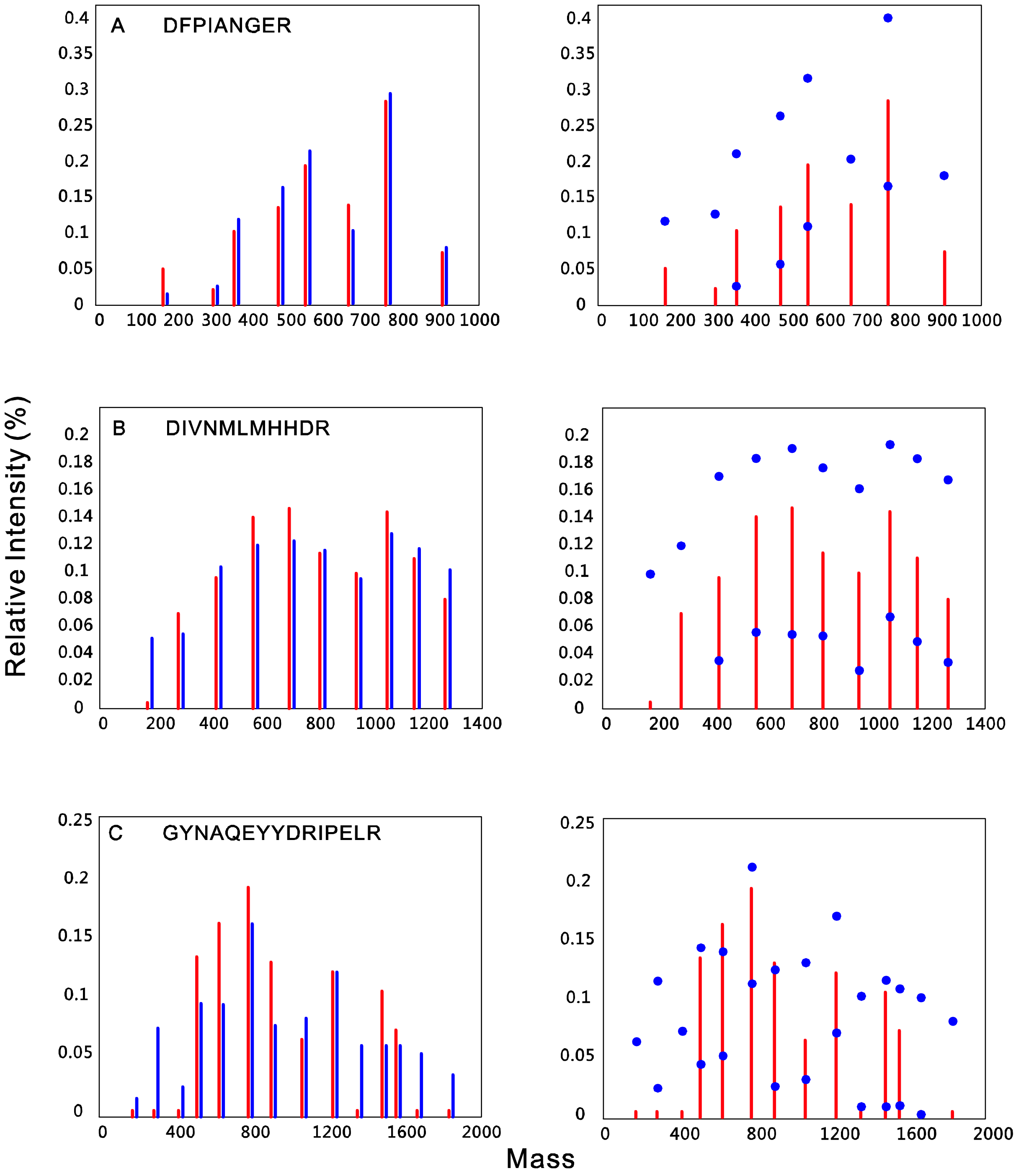

Figure 1-A: Mobile peptide with residue Asp and Pro. Similarity score: 9.32.
Figure 1-B: Partial-mobile peptide with Asp. Similarity score: 8.19.
Figure 1-C: Non-mobile peptide with Asp and Pro. Similarity score: 14.64.
Experimental spectra are log-transformed and indicated in red. Predicted spectra are indicated in blue. Both types of spectra are subject to total ion current normalization. Blue dots indicate the interval [mean intensity – SD, mean intensity + SD] within which intensities of the ions are supposed to lie.
